# Effect of the addition of rosiglitazone to metformin or sulfonylureas versus metformin/sulfonylurea combination therapy on ambulatory blood pressure in people with type 2 diabetes: A randomized controlled trial (the RECORD study)

**DOI:** 10.1186/1475-2840-7-10

**Published:** 2008-04-24

**Authors:** Michel Komajda, Paula Curtis, Markolf Hanefeld, Henning Beck-Nielsen, Stuart J Pocock, Andrew Zambanini, Nigel P Jones, Ramon Gomis, Philip D Home, The RECORD Study Group

**Affiliations:** 1 Université Pierre et Marie Curie Paris 6; Assistance Publique Hôpitaux de Paris, Hôpital Pitié-Salpêtrière, Paris, France; 2 GlaxoSmithKline Pharmaceuticals, Greenford, UK; 3 Zentrum für Klinische Studien Forschungsbereich Endokrinologie und Stoffwechsel, Dresden, Germany; 4 Department of Endocrinology & Metabolism, Odense, Denmark; 5 London School of Hygiene and Tropical Medicine, UK; 6 GlaxoSmithKline Pharmaceuticals, Harlow, UK; 7 Hospital Clinic, Barcelona University, Spain; 8 Newcastle Diabetes Centre and Newcastle University, UK

## Abstract

**Background:**

Hypertension and type 2 diabetes are common co-morbidities. Preliminary studies suggest that thiazolidinediones reduce blood pressure (BP). We therefore used ambulatory BP to quantify BP lowering at 6–12 months with rosiglitazone used in combination with metformin or sulfonylureas compared to metformin and sulfonylureas in people with type 2 diabetes.

**Methods:**

Participants (n = 759) in the multicentre RECORD study were studied. Those taking metformin were randomized (open label) to add-on rosiglitazone or sulfonylureas, and those on sulfonylurea to add-on rosiglitazone or metformin.

**Results:**

24-Hour ambulatory BP was measured at baseline, 6 months and 12 months. At 6 and 12 months, reductions in 24-hour ambulatory systolic BP (sBP) were greater with rosiglitazone versus metformin (difference at 6 months 2.7 [95% CI 0.5–4.9] mmHg, p = 0.015; 12 months 2.5 [95% CI 0.2–4.8] mmHg, p = 0.031). Corresponding changes for ambulatory diastolic BP (dBP) were comparable (6 months 2.7 [95% CI 1.4–4.0] mmHg, p < 0.001; 12 months 3.1 [95% CI 1.8–4.5] mmHg, p < 0.001). Similar differences were observed for rosiglitazone versus sulfonylureas at 12 months (sBP 2.7 [95% CI 0.5–4.9] mmHg, p = 0.016; dBP 2.1 [95% CI 0.7–3.4] mmHg, p = 0.003), but differences were smaller and/or not statistically significant at 6 months (sBP 1.5 [95% CI -0.6 to 3.6] mmHg, p = NS; dBP 1.3 [95% CI 0.0–2.5] mmHg, p = 0.049). Changes in BP were not accompanied by compensatory increases in heart rate, did not correlate with basal insulin sensitivity estimates and were not explained by changes in antihypertensive therapy between the various strata.

**Conclusion:**

When added to metformin or a sulfonylurea, 12-month treatment with rosiglitazone reduces ambulatory BP to a greater extent than when metformin and a sulfonylurea are combined.

**Trial registration:**

NCT00379769

## Background

Cardiovascular disease accounts for the majority of mortality and morbidity associated with type 2 diabetes [1-3]. Elevated blood pressure (BP) is a major cardiovascular risk factor in type 2 diabetes. Lowering BP has been shown to reduce the risk of cardiovascular complications in these people [4-7], and is particularly important in diabetes care due to the prevention of retinopathy and nephropathy [4,8,9]. Thus, BP reduction is both clinically and economically more effective in people with diabetes [10], and guidelines now recommend lower BP treatment thresholds and targets than for non-diabetic people [5-7,11].

Rosiglitazone is a PPAR-γ (peroxisome proliferator-activated receptor-gamma) agonist that has been shown to improve insulin sensitivity, resulting in improved glycaemic control in people with type 2 diabetes [12-14]. Thiazolidinediones have been shown to exert beneficial effects on inflammation or coagulation markers and on lipoprotein profile *in vivo *[15-17]. Moreover, it was observed recently that a thiazolidinedione slowed the progression of carotid intima-media thickness, a validated surrogate marker of atherosclerosis and cardiovascular risk, when compared with glimepiride in type 2 diabetic patients [18]. Finally, these compounds have also been reported to lower BP in animal models [19], people with impaired glucose tolerance [20], people with type 2 diabetes with and without hypertension [21-27] and non-diabetic hypertensives [28]. However, these BP studies have significant limitations, being uncontrolled observations [25,26,28] of small sample size [20,21,23-28] and/or short treatment duration [20,23-25,27,28].

There was thus a need for a large, adequate duration, prospectively defined and actively controlled study from which the effect of rosiglitazone on BP could be adequately assessed. RECORD (Rosiglitazone Evaluated for Cardiac Outcomes and Regulation of glycaemia in Diabetes), a study of combination oral therapies, was a useful environment in which to do this, the design allowing comparison of rosiglitazone against both metformin and sulfonylureas [29]. Ambulatory BP measurement (ABPM), while too cumbersome to apply in every centre participating in the RECORD study, offered the chance of more accurate and precise assessments than performed previously. The present paper therefore reports the results of 12-month ABPM conducted as a prospectively designed study of a subset of participants within the RECORD trial [29].

## Methods

The design of the RECORD study has been described in detail elsewhere [29].

### Participants

RECORD involves 330 study centres in 23 countries in Europe and Australasia. People with type 2 diabetes (n = 4458) inadequately controlled on metformin or sulfonylurea monotherapy were enrolled. The pre-specified 12-month ABPM study was conducted at 128 centres in 16 countries in Europe. At these centres, people were invited to participate in the ABPM study at the time of enrolment into the main study. Enrolment continued until target entry numbers with a valid baseline ABPM record were reached in each group. Eligible participants had type 2 diabetes as defined by the 1999 World Health Organization criteria [30], were aged 40–75 years, with a body mass index of > 25.0 kg/m^2 ^and HbA_1c _7.1–9.0%, on maximum permitted or tolerated doses of metformin or a sulfonylurea (glibenclamide [glyburide], glimepiride or gliclazide) at study entry. Individuals were not to be included if their clinic BP was > 180/105 mmHg.

The ABPM study protocol was approved by ethics review according to local laws/customs and was carried out in accordance with the Declaration of Helsinki. Written informed consent was obtained before beginning any protocol-specific procedure.

### Study design

This study is a multicentre, randomized, open-label, comparative, parallel-group trial [29]. Eligible participants continued to take their current glucose-lowering drug (metformin or sulfonylurea) and entered a 4-week run-in period of reinforcement of lifestyle education, followed by concealed randomization. Treatment allocation was stratified for current glucose-lowering medication. Those taking a sulfonylurea were randomized to additional rosiglitazone or metformin, and those taking metformin to additional rosiglitazone or a sulfonylurea (glibenclamide, gliclazide or glimepiride, according to local practice). Throughout the study, participants were treated to a target HbA_1c _of ≤ 7.0%. If HbA_1c _rose above 7.0% at any point after 8 weeks of randomized treatment, the dose of the study medication was increased to a maximum of 4 mg rosiglitazone twice daily, 2550 mg/day metformin, 15 mg/day glibenclamide (or equivalent), 240 mg/day gliclazide or 4 mg/day glimepiride. If HbA_1c _was ≥ 8.5% (confirmed) on the maximum tolerated dose for at least 8 weeks, a third glucose-lowering agent was added and their data censored from that point onwards.

Any antihypertensive medication in use before randomization could be continued during the study. For participants whose BP was subsequently judged by their physician to require additional medication, it was recommended to modify treatment in accordance with the IDF Type 2 Diabetes European Policy Group guidelines [31].

### Ambulatory blood pressure monitoring

Ambulatory BP was measured using a Spacelabs 90207 device (Spacelabs, Redmond, WA, USA) during the week prior to randomization and at 6 and 12 months [32]. The device recorded diastolic and systolic BP (dBP and sBP) and heart rate every 20 minutes from 08:00 to 22:00 h ('day-time') and every hour from 22:00 to 08:00 h ('night-time'), such that approximately 52 readings were taken during the 24-hour assessment. Weighted mean 24-hour, daytime and night-time BP and heart rate were calculated.

To be used for the analysis, the 24-hour ABPM had to span a minimum of 24 hours, and have no more than two non-consecutive day-time hours with fewer than two valid readings, and no more than two non-consecutive night-time hours with no valid readings. The validity of recordings was determined by a third party (Biomedical Systems, Brussels, Belgium), blind to treatment allocation.

### Insulin sensitivity, body weight and adverse events

Homeostasis model assessment estimates of insulin sensitivity (HOMA%S) were calculated using the HOMA Calculator (version 2.2; Oxford Trials Unit, Oxford, UK) [33]. The inputs to the HOMA model, fasting plasma glucose and serum insulin were assayed at a central laboratory (Quest Diagnostics, Heston, UK) as previously described [29]. Body weight was assessed at baseline and all six follow-up visits.

Although the RECORD study is ongoing to 2008, some preliminary efficacy and safety-related data (including fluid retention) were published urgently in 2007, following publication of a meta-analysis of some rosiglitazone studies [34,35]. Some other outcome data for rosiglitazone have become available at FDA Advisory Committee hearings. However, a full safety analysis from RECORD will await study completion [36].

### Statistical analysis

The primary efficacy measure for this ABPM sub-study was the change from baseline in 24-hour ambulatory dBP after 6 months between rosiglitazone and comparator in the two background therapy groups. Hence, only those with a valid ambulatory baseline assessment are considered. A sample size of 141 participants per treatment group (564 in total) was estimated to give an 80% power of detecting a 2 mmHg difference in dBP between treatment groups, assuming a standard deviation of 6 mmHg for the change from baseline and a two-sided alpha = 0.05.

Changes from baseline in mean 24-hour, day- and night-time ambulatory dBP and sBP, and heart rate were analysed using repeated measures at 6 and 12 months for the modified intent-to-treat (ITT) population (all randomized, treated and with at least one data point post-randomization). The model included terms for age, gender, presence of hypertension, treatment and baseline by visit interaction, and employed an unstructured covariance matrix to model the within-patient variability for each treatment group. Presence of hypertension was taken as average baseline daytime ABPM > 135/85 mmHg or prior diagnosis of hypertension. No adjustment was made for the confounding effects due to new antihypertensive medication during follow-up, as this cannot be done reliably.

Antihypertensive medication use (number of agents and class) was summarized at baseline and at end of follow-up in all treatment groups. In order to take into account changes occurring in antihypertensive therapy, the time-course for first introducing new/increased antihypertensive medication was estimated using the Kaplan-Meier method, and treatment groups compared using Log rank tests.

To assess the day-night interaction, the differences (day-night) for dBP and sBP, and heart rate were analysed using the same methodology as for other BP measures. This methodology was also used for analyses of body weight. The relationship between changes from baseline in BP and each of log-transformed HOMA%S and body weight by 12 months was explored by scatter plots and correlation coefficients (Pearson and Spearman rank). All analyses were adjusted for baseline measurements to correct for any baseline imbalances between treatment groups. All significance tests and confidence intervals were two sided and performed or constructed at the 5% significance level. Analyses were conducted using SAS for Windows (version 8.2; SAS Institute, Cary, NC, USA).

## Results

A total of 926 people were randomized (ABPM study), of whom 759 had a valid baseline ABPM profile. Of these, 668 had at least one valid post-randomization ABPM profile (545 at both 6 and 12 months, 88 at 6 months only, 35 at 12 months only), forming the modified ITT population. Baseline characteristics are given in Table 1.

**Table 1:** Clinical characteristics of the population studied

	*Background metformin*		*Background sulfonylurea*	
		
	*+ rosiglitazone*	*+ sulfonylurea*	+ *rosiglitazone*	+ *metformin*
Participants (n)	176	165	160	167
Age (yr)	57 ± 8	57 ± 8	60 ± 8	58 ± 8
Male (n (%))	80 (45)	86 (52)	83 (52)	77 (46)
Europid (n (%))	175 (> 99)	165 (100)	160 (100)	167 (100)
Body weight (kg)	92 ± 17	94 ± 16	86 ± 13	84 ± 16
BMI (kg/m^2^)	33 ± 5	32 ± 5	31 ± 4	30 ± 5
Time from diabetes diagnosis (yr)	5.7 ± 3.8	5.9 ± 3.6	7.7 ± 5.1	7.1 ± 5.1
HbA_1c _(%)	7.8 ± 0.6	7.8 ± 0.8	7.9 ± 0.7	8.0 ± 0.8
Fasting plasma glucose (mmol/l)	9.2 ± 2.1	9.7 ± 2.3	10.0 ± 2.4	10.2 ± 2.4
Homeostasis model assessment %S (%)	68 (42, 106)	64 (42, 108)	64 (47, 88)	63 (42, 93)
Hypertension* (n (%))	145 (82)	138 (84)	139 (87)	141 (84)
Treated with antihypertensive drugs	119 (68)	112 (68)	104 (65)	114 (68)
Ambulatory systolic BP (mmHg)				
24-hour	132 ± 14	134 ± 16	132 ± 13	132 ± 14
Day-time	136 ± 14	138 ± 15	135 ± 13	136 ± 14
Night-time	126 ± 15	128 ± 18	126 ± 14	127 ± 15
Ambulatory diastolic BP (mmHg)				
24-hour	78 ± 8	78 ± 9	76 ± 8	76 ± 8
Day-time	81 ± 8	82 ± 10	79 ± 8	79 ± 8
Night-time	72 ± 9	73 ± 10	71 ± 9	71 ± 9
Ambulatory heart rate (beat/min)				
24-hour	76 ± 10	75 ± 9	74 ± 11	75 ± 10
Day-time	81 ± 11	80 ± 10	79 ± 12	78 ± 11
Night-time	70 ± 9	69 ± 9	68 ± 10	69 ± 9

Number (percent) or mean ± SD, or median (IQR).

*Medical history or average baseline day-time ABPM > 135/85 mmHg

Approximately half of the participants were male and all but one was Europid. Within stratum the randomized groups were well matched, but the background metformin stratum was younger, more overweight and had shorter duration of diabetes than the sulfonylurea stratum. The presence of microalbuminuria at baseline was low in all four treatment groups. Eighty-four percent of participants had hypertension at baseline, already diagnosed (73%) or identified by the baseline ABPM (11%). Antihypertensive medication was being taken at baseline by 449 (67%) participants (single agent 207 participants [31%], two drugs 159 [24%], three or more drugs 83 [12%]). The distribution of class of antihypertensive treatment and number of agents was very similar in all four treatment groups (Table 2).

**Table 2:** Blood pressure lowering medication at baseline and end of follow-up

	*Background metformin*		*Background sulfonylurea*	
		
	+ *rosiglitazone*	+ *sulfonylurea*	+ *rosiglitazone*	+ *metformin*
Modified ITT population (n)	176	165	160	167
Type of medication (n (%))				
Baseline				
Any BP medication	119 (68)	112 (68)	104 (65)	114 (68)
ACEi/ARB	91 (52)	83 (50)	80 (50)	88 (53)
β-blocker	44 (25)	42 (25)	34 (21)	43 (26)
CCB	37 (21)	33 (20)	28 (17)	36 (22)
Diuretics	28 (16)	34 (21)	26 (16)	38 (23)
End of follow-up*				
Any BP medication	131 (74)	124 (75)	119 (74)	121 (72)
ACEi/ARB	99 (56)	93 (56)	89 (56)	92 (55)
β-blocker	51 (29)	53 (32)	40 (25)	46 (27)
CCB	47 (27)	39 (24)	32 (20)	40 (24)
Diuretics	48 (27)	39 (24)	34 (21)	41 (25)
Number of drug classes (n (%))				
Baseline				
0	57 (32)	53 (32)	56 (35)	53 (32)
1	62 (35)	47 (28)	51 (32)	47 (28)
2	34 (19)	45 (27)	37 (23)	43 (26)
3	17 (10)	15 (9)	14 (9)	20 (12)
> 3	6 (3)	5 (3)	2 (1)	4 (2)
End of follow-up				
0	45 (25)	41 (25)	41 (26)	46 (27)
1	57 (32)	47 (28)	58 (36)	45 (27)
2	34 (19)	47 (28)	41 (26)	50 (30)
3	27 (15)	22 (13)	15 (9)	19 (11)
> 3	13 (7)	8 (5)	5 (3)	7 (4)

Data are n or n (%).

ACEi, ACE inhibitor; ARB, angiotensin receptor blocker; CCB, calcium channel blocker.

*End of follow-up refers to time of stopping dual combination therapy or 12 months, whichever is sooner

After 12 months, 96% of the participants in the modified ITT population were continuing to take their allocated dual oral glucose-lowering therapy. More participants in the metformin+rosiglitazone and sulfonylurea+rosiglitazone groups ceased dual combination treatment (8% and 5%, respectively) than in non-rosiglitazone groups (< 1% for both), the majority by progression to triple therapy (6% and 4%, respectively).

Changes in antihypertensive medication at end of follow-up were similar in all groups (Table 2). Antihypertensive medication was started in the background metformin stratum in 27 (8%) participants (rosiglitazone 13; sulfonylurea 14), additional drugs were taken by 55 (16%) (rosiglitazone 31; sulfonylurea 24), and an increased dose in 18 (5%) (rosiglitazone 9; sulfonylurea 9). The same matched intensification was observed in the background sulfonylurea stratum: 24 participants (7%) started treatment (rosiglitazone 15; metformin 9), 39 (12%) added a new drug (rosiglitazone 19; metformin 20), and 15 (5%) increased the dose (rosiglitazone 7; metformin 8).

The time-course for first introducing new/increased antihypertensive medication during follow-up was similar for rosiglitazone-treated patients and the respective control groups (Log rank test p-value both > 0.50). Kaplan-Meier estimates of the proportion who introduced new/increased antihypertensive medication by 12 months were 30.1% (95% CI 23.2–37.0) and 28.7% (95% CI 21.6–35.7) of the background metformin participants on rosiglitazone and sulfonylurea, respectively, and 26.4% (95% CI 19.3–33.5) and 21.5% (95% CI 14.8–28.2) of background sulfonylurea participants using rosiglitazone and metformin, respectively.

### 24-hour ambulatory blood pressure

**Figure 1:**
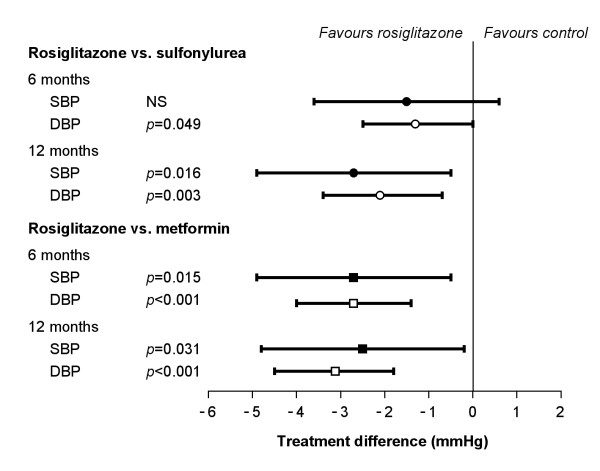
Model-adjusted mean difference in ABPM for rosiglitazone compared to sulfonylurea and to metformin in combination therapy.

The majority of participants had at least 85% of valid readings during the 24-hour ABPM assessment. For rosiglitazone added to background sulfonylurea, the reduction in 24-hour sBP was significantly greater at both 6 (-3.8 mmHg) and 12 (-3.8 mmHg) months than with metformin added to sulfonylurea (-1.2 and -1.3 mmHg) (6 months, p = 0.015; 12 months, p = 0.031) (Figure 1 and Table 3). Reductions in 24-hour dBP were also statistically significantly greater at both 6 and 12 months with rosiglitazone added to sulfonylurea (-3.1 and -3.7 mmHg) than with metformin added to sulfonylurea (-0.4 and -0.6 mmHg; both p < 0.001).

**Table 3:** Change from baseline in 24-h ambulatory BP and heart rate at 6 and 12 months

	*Background metformin*			*Background sulfonylurea*		
		
	+ *rosiglitazone *(n = 176)	+ *sulfonylurea *(n = 165)	*difference (95% CI), p-value*	+ *rosiglitazone *(n = 160)	+ *metformin *(n = 167)	*difference (95% CI), p-value*
ystolic BP change (mmHg)						
6 months	-3.1 (-4.8, -1.4)	-1.6 (-3.5, + 0.3)	-1.5 (-3.6, 0.6), NS	-3.8 (-5.8, -1.8)	-1.2 (-3.0, + 0.7)	-2.7 (-4.9, -0.5), 0.015
12 months	-4.9 (-6.7, -3.2)	-2.2 (-4.2, -0.3)	-2.7 (-4.9, -0.5), 0.016	-3.8 (-5.9, -1.8)	-1.3 (-3.3, + 0.7)	-2.5 (-4.8, -0.2), 0.031
Diastolic BP change (mmHg)						
6 months	-2.8 (-3.8, -1.9)	-1.6 (-2.7, -0.5)	-1.3 (-2.5, -0.0), 0.049	-3.1 (-4.2, -2.0)	-0.4 (-1.6, + 0.7)	-2.7 (-4.0, -1.4), < 0.001
12 months	-3.8 (-4.9, -2.7)	-1.7 (-2.9, -0.5)	-2.1 (-3.4, -0.7), 0.003	-3.7 (-4.9, -2.5)	-0.6 (-1.7, + 0.6)	-3.1 (-4.5, -1.8), < 0.001
Heart rate change (beat/min)						
6 months	-0.4 (-1.6, + 0.8)	-0.1 (-1.3, + 1.0)	-0.3 (-1.7, 1.1), NS	-0.7 (-1.9, + 0.5)	1.3 (+ 0.1, + 2.6)	-2.0 (-3.4, -0.6), 0.006
12 months	-0.9 (-2.2, + 0.4)	0.0 (-1.3, + 1.3)	-0.9 (-2.5, 0.7), NS	-0.9 (-2.3, + 0.5)	1.7 (+ 0.3, + 3.1)	-2.6 (-4.2, -1.0), 0.002

Modified ITT population; data are model-adjusted mean (95% CI), or number

At 12 months, the reduction in 24-hour sBP was significantly greater (p = 0.016) for rosiglitazone added to metformin (-4.9 mmHg) than for sulfonylurea added to metformin (-2.2 mmHg) (Figure 1 and Table 3). Diastolic BP at 12 months was also reduced to a greater extent by rosiglitazone added to metformin (-3.8 mmHg) than by sulfonylurea added to metformin (-1.7 mmHg; p = 0.003). At 6 months, both for sBP and dBP, the changes from baseline and difference between the treatment groups were less pronounced (sBP, p = NS; dBP, p = 0.049).

Data analysis for the sub-population with hypertension at baseline (84% of the study population) gave very similar results to those of the whole population (data not shown).

No sizeable correlation was found at 12 months between change in 24-hour ABPM and change in HOMA%S in any of the rosiglitazone treatment groups (r= -0.03 to -0.20) (data not shown). In addition, no sizeable correlation was found at 12 months between change in 24-hour ABPM and body weight change (r= -0.14 to 0.16; data not shown).

Similar increases in body weight from baseline to 12 months were observed in both arms of the metformin stratum (rosiglitazone: +1.9 kg [95% CI 1.3–2.6]; sulfonylurea: +1.5 kg [95% CI 1.0–2.1]; difference: 0.4, p = NS). In the sulfonylurea stratum there was a significant increase in body weight with rosiglitazone compared to a slight decrease with metformin (rosiglitazone: +2.2 kg [95% CI 1.6–2.7]; metformin: -1.1 kg [95% CI -1.5 to -0.6]; difference: 3.3, p < 0.0001).

### Diurnal blood pressure contrasts

In general, the day- and night-time analyses were consistent with the 24-hour findings, with all the BP reductions on rosiglitazone at 12 months numerically greater than for comparator metformin and sulfonylurea arms, and in the majority of these sub-analyses statistically significant (Table 4).

**Table 4:** Contrast between day- and night-time effects of rosiglitazone on ambulatory BP at 12 months

	*Background metformin*	*Background sulfonylurea*
	
	*rosiglitazone vs sulfonylurea (difference (95% CI), p-value)*	*rosiglitazone vs metformin (difference (95% CI), p-value)*
Systolic BP (mmHg)		
24-hour	-2.7 (-4.9, -0.5), 0.016	-2.5 (-4.8, -0.2), 0.031
Day-time	-3.3 (-5.6, -1.0), 0.004	-1.6 (-3.9, 0.9), NS
Night-time	-1.5 (-3.9, 1.0), NS	-4.0 (-6.6, -1.3), 0.004
Diastolic BP (mmHg)		
24-hour	-2.1 (-3.4, -0.7), 0.003	-3.1 (-4.5, -1.8), < 0.001
Day-time	-2.5 (-3.9, -1.0), 0.001	-2.5 (-3.9, -1.0), 0.001
Night-time	-1.3 (-2.9, 0.3), NS	-4.0 (-5.7, -2.4), < 0.001

For each contrast, the confidence intervals of the day- and night-time differences overlap. However, the effects of rosiglitazone compared with metformin tended to be larger during the night than during the day (day-night difference: sBP, p = 0.018; dBP, p = 0.020), while the effects compared to sulfonylurea tended to be larger or no different during the day (sBP, p = 0.052; dBP, p = NS).

The day-night profiles at 6 months were similar to those observed at 12 months (data not shown).

### Ambulatory heart rate

The greater reductions in BP in the rosiglitazone groups were not accompanied by a compensatory increase in ambulatory heart rate (Table 3). Heart rate did increase in the group in which metformin was added to sulfonylurea, such that adjusted heart rate changes from baseline at 6 and 12 months were statistically significantly lower with rosiglitazone (Table 3).

## Discussion

### Blood pressure lowering effect of rosiglitazone

The study was designed to provide the first large, adequate duration, prospectively defined and controlled trial in which the effect of rosiglitazone on BP profile in people with type 2 diabetes could be accurately determined. Some long-term data are available for pioglitazone, another PPAR-γ agonist, but those data were not actively controlled, are based on clinic measurements and were gained in a sub-population of people with overt cardiovascular disease [37].

Our primary finding was that rosiglitazone added to either metformin or to a sulfonylurea reduced ambulatory sBP and dBP and this effect was greater than that observed with the standard diabetes combination treatment of metformin and a sulfonylurea. The magnitude of the reductions in BP is consistent with that reported in previous smaller, uncontrolled or shorter duration studies [20-28]. The reductions are smaller than those achieved with main-line antihypertensive agents but, importantly, were achieved in a population already receiving appropriate care for BP management, and therefore not markedly hypertensive. The changes in antihypertensive therapy (number of agents and dosage) and time-course for first introducing new/increased therapy during follow-up were similar for rosiglitazone-treated patients and respective control groups, and are therefore unlikely to have caused bias or explain the findings.

Rosiglitazone reduced sBP and dBP compared with active controls in both day- and night-time periods in most, but not all, of the treatment comparisons, with an inconsistent pattern by background therapy (added to metformin or sulfonylurea). This suggests that, while the sample size proved sufficient to investigate the 24-hour measurement, there was insufficient power to interrogate effects completely reliably when the data were divided between day and night. Nevertheless, the observed data portray a greater effect of rosiglitazone versus metformin during the night and a greater effect versus sulfonylurea during the day. This finding should be taken as observational and needs confirmation.

### Potential mechanism of the effect on blood pressure

The mechanism by which rosiglitazone reduces BP remains unclear.

1) Previous small studies have reported an association between reductions in BP seen with rosiglitazone and its effects on insulin sensitivity measured by euglycaemic-hyperinsulinaemic clamp [28] or estimated by HOMA [21,26]. However, the lack of correlation between changes in HOMA-estimated insulin sensitivity and ambulatory BP changes in this much larger sample suggests that the mechanisms by which rosiglitazone exerts these two effects are likely to be independent of each other. This lack of correlation is in line with the fact that some beneficial effects of thiazolidinediones on inflammation or atherosclerosis markers have been shown to be independent from blood glucose control and attributed to PPAR-γ [38].

2) Improvement in endothelial function might be a contributor to BP reduction since improvements in endothelial function have been attributed to rosiglitazone as determined by improvements in forearm blood flow [23,39], reductions in asymmetric dimethylarginine concentrations [40] and improved arterial function and elasticity [41].

3) A direct vascular effect of thiazolidinediones has been demonstrated in vascular smooth muscle *in vitro *through a blockade of calcium uptake. This inhibition of inward Ca^2+ ^current through L-type channels in vascular smooth muscle might result in a BP-lowering effect through a vasodilatory effect [42-44]. Since arterial tone is influenced by the activation of calcium-dependent potassium channels in several vascular beds, the blocking effect of sulfonylurea agents such as glibenclamide on these channels might result in a vasoconstrictive effect, leading to increased BP [45]. We indeed observed a greater BP-lowering effect when rosiglitazone was added to sulfonylurea than when it was added to metformin.

4) Reductions in insulin resistance at the endothelial cell level, leading to an improvement in endothelial function, could be postulated as a contributor to the BP reduction, even if it now seems unlikely to be related to a whole body effect on insulin sensitivity. One such mechanism might be through antioxidant properties [46].

5) Other potential mechanisms that have been explored include a down regulation of sympathetic nerve activity [27] and effects secondary to the decrease in plasma non-esterified fatty acids or to the increase in insulin sensitivity. Rosiglitazone has also been shown to down regulate the renin-angiotensin system in human subcutaneous adipose tissue [47] and it has been proposed that this effect may contribute to its BP-lowering activity. The BP lowering was observed without significant increase in heart rate, suggesting that a vasodilatory effect is not the predominant mechanism.

### Potential implication for cardiovascular risk

Prospective observational studies, such as that performed within the UK Prospective Diabetes Study [48], suggest that in people with type 2 diabetes there was a linear relationship with no threshold between BP reduction and cardiovascular risk, and have thus concluded that any reduction of raised BP is likely to have benefit. The observed reductions in sBP and dBP were in the range of 2–3 mmHg. Such a reduction in BP has been observed in several large BP trials comparing active treatments and placebo, or aggressive versus less aggressive strategies, and was associated with a significant improvement in cardiovascular outcome [49,50]. To what extent changes in BP with thiazolidinediones translate into a significant decrease in cardiovascular risk remains unclear. In a report to an FDA Advisory Committee, the manufacturer of rosiglitazone reported a significant decrease in stroke for integrated analysis of early short-term trials (HR 0.48 [0.23–0.98]) and a non-significant change for the RECORD interim analysis (HR 0.76 [0.47–1.23]) [36].

It is noteworthy that in a study using carotid intima-media thickness, a recognized surrogate marker for cardiovascular risk, the effect of pioglitazone seemed numerically greater (although not significant statistically) in the subgroup of patients with higher BP levels at baseline [18]. The number of cardiovascular events was limited in this study owing to the size and duration of the study, but was smaller in the thiazolidinedione arm.

In a large outcome trial of more than 5000 patients with type 2 diabetes and evidence of macrovascular disease, pioglitazone did not significantly reduce the composite primary end point but did significantly reduce the risk of the main secondary end point, including all-cause mortality, myocardial infarction or stroke, and also reduced the recurrence of myocardial infarction in the subgroup of patients with previous myocardial infarction [37,51].

A recent meta-analysis including 42 trials, many of which were short term, suggested that rosiglitazone was associated with a significant increase in the risk of myocardial infarction [34]. These results were, however, not confirmed by the interim analysis of the major cardiovascular events occurring in the RECORD trial [35]. This analysis, after 3.75 years of follow-up, was inconclusive regarding the effect of rosiglitazone on the risk of death or hospitalization from cardiovascular causes and was insufficient to determine whether the drug was associated with an increase in the risk of myocardial infarction. Observational studies have also shown conflicting results [52,53]. Overall, these results indicate that the impact of thiazolidinediones and particularly of rosiglitazone on cardiovascular outcome is unclear.

Combination oral-agent therapy to achieve guideline-advocated targets for blood glucose control is becoming commonplace. The setting for the present study, in which rosiglitazone is being used as part of a combination treatment regimen, thus has the advantage of being particularly clinically relevant. The patient demography and baseline assessments were very similar in this sub-study group and the total RECORD population of 4458 individuals [29], suggesting that the selection of sites and enrolment by specific invitation for the ABPM study did not lead to a subgroup which was unrepresentative of the study as a whole. Other strengths of this study include its large population, 12-month treatment duration, use of 24-hour ambulatory BP assessment (as opposed to sitting office measurements) and the consistency of the rosiglitazone effects in the two background treatment strata. Both the glucose-lowering study drugs and background antihypertensive drugs were used in line with prevailing clinical practice without the artificial restrictions that characterize many clinical trials.

### Limitations

However, the study does have a number of weaknesses. This was an open-label study, which could have undermined the concealed randomization if appreciable numbers of participants had withdrawn after being told to which treatment group they had been allocated. However, only one subject withdrew after randomization before starting treatment. In order to reduce the potential for bias on the primary end point, the decision on the validity of all ambulatory BP readings was made observer blind by an independent third party. Another potential limitation was that background antihypertensive therapies could be modified during the study, but increases in dose and addition of new agents and the time course of these events were well balanced across all study treatment groups. The treatment algorithm for managing unacceptably high levels of glucose control was by necessity asymmetrical and thus ambulatory BP assessments were censored after these transitions from dual oral therapy. In the early stages of the study, more patients stopped dual oral therapy in the rosiglitazone-containing arms than those on metformin plus sulfonylurea, which might have introduced a patient or physician preference bias given the open-label nature of the study. However, the absolute numbers involved were small.

## Conclusion

This sub-study has demonstrated that rosiglitazone, added to either metformin or to a sulfonylurea, reduces ambulatory BP and that this effect, following 12-month treatment, is greater than that observed with the standard glucose-lowering combination of metformin and a sulfonylurea. Whether the reduction in BP observed with this compound translates into improved cardiovascular outcome needs further evaluation.

## Abbreviations

ABPM: ambulatory blood pressure measurement; BP: blood pressure; dBP: diastolic blood pressure; HOMA%S: homeostasis model assessment estimates of insulin sensitivity; ITT: intent-to-treat; PPAR-γ: peroxisome proliferator-activated receptor-gamma; RECORD: Rosiglitazone Evaluated for Cardiovascular Outcomes and Regulation of Glycaemia in Diabetes; sBP: systolic blood pressure.

## Competing interests

The RECORD study was sponsored by GlaxoSmithKline, the manufacturer of rosiglitazone. Members of the Steering Committee, Data Safety and Monitoring Board, and Clinical Endpoints Committee, or their institutions, are remunerated for their time and expenses, and some are similarly engaged on other consultative, research and teaching activities with the sponsor. Local investigators and/or their institutions are paid fees per participant for study activities; some are involved with other activities of the sponsor. M. Komajda, M. Hanefeld, H. Beck-Nielsen, S. Pocock, R. Gomis and P.D. Home are members of the RECORD Steering Committee. P. Curtis, A. Zambanini and N. Jones are employees of GlaxoSmithKline.

## Authors' contributions

MK, PC, MH, HB-N, SJP, NPJ, RG and PDH were involved in the design and coordination of the study. PC, SJP and AZ carried out the data analysis. All authors were involved in the development of the manuscript. All authors read and approved the final manuscript.
